# The Structure, Function, and Physiology of the Fetal and Adult Acetylcholine Receptor in Muscle

**DOI:** 10.3389/fnmol.2020.581097

**Published:** 2020-09-08

**Authors:** Hakan Cetin, David Beeson, Angela Vincent, Richard Webster

**Affiliations:** ^1^Department of Neurology, Medical University of Vienna, Vienna, Austria; ^2^Nuffield Department of Clinical Neurosciences, University of Oxford, Oxford, United Kingdom

**Keywords:** fetal acetylcholine receptor, adult acetylcholine receptor, ion channel, myasthenia, neuromuscular junction, muscle development

## Abstract

The neuromuscular junction (NMJ) is a highly developed synapse linking motor neuron activity with muscle contraction. A complex of molecular cascades together with the specialized NMJ architecture ensures that each action potential arriving at the motor nerve terminal is translated into an action potential in the muscle fiber. The muscle-type nicotinic acetylcholine receptor (AChR) is a key molecular component located at the postsynaptic muscle membrane responsible for the generation of the endplate potential (EPP), which usually exceeds the threshold potential necessary to activate voltage-gated sodium channels and triggers a muscle action potential. Two AChR isoforms are found in mammalian muscle. The fetal isoform is present in prenatal stages and is involved in the development of the neuromuscular system whereas the adult isoform prevails thereafter, except after denervation when the fetal form is re-expressed throughout the muscle. This review will summarize the structural and functional differences between the two isoforms and outline congenital and autoimmune myasthenic syndromes that involve the isoform specific AChR subunits.

## Structural Determinants of Functional Differences Between Fetal and Adult AChRs

**FIGURE 1:**
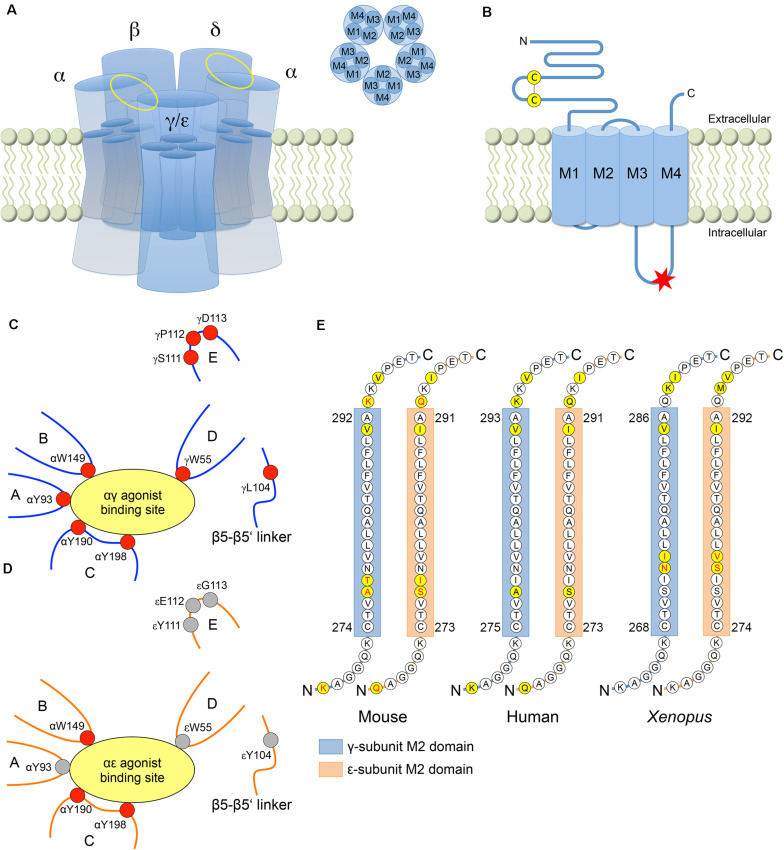
Structural differences between the γ- and ε-subunit. **(A)** AChR consisting of five subunits with the fetal specific γ-subunit or the adult specific ε-subunit. Each subunit has four helical transmembrane domains M1-M4. The αγ/αε and αδ agonist binding sites are located between the corresponding subunits (yellow ellipses). **(B)** AChR subunit with N-terminal extracellular domain containing the Cys-loop, four transmembrane domains and a cytoplasmic loop domain between M3 and M4. In human, a consensus sequence for phosphorylation by protein kinase A (RRXSX, where X is any amino acid) is only present in the M3-M4 loop of the ε-subunit (red asterisk) without any corresponding sequence in the γ-subunit (residues 370–374 in the ε-subunit, i.e., RRASS). **(C)** At the αγ binding site, the favorable free energy resulting from the change of ACh affinities upon transformation of the AChR from the resting into the open-channel state is provided by the five aromatic core residues αY93, αW149, αY190, αY198, and γW55 from discontinuous sections of the primary sequence that rearrange close to the binding site upon agonist binding. The relative conformation of the aromatic core is stabilized by four other residues (γL104, γS111, γP112, and γD113) increasing the affinity of the αγ binding site to the agonist. **(D)** At the αε binding site, acetylcholine binding energy is provided by only three (instead of five) aromatic core residues (αW149, αY190, and αY198), resulting in a lower affinity to the agonist (with residues contributing to acetylcholine binding energy in red). **(E)** The majority of the amino acid residues of the pore forming M2 domain is highly conserved across paralogs and species. The γ- and ε-subunits differ at only three amino acid residues in mouse and *Xenopus*, and at two residues in human (yellow circles). The AChR isoform specific conductance was shown to be determined by amino acid residues within the M2 domain (in mouse and *Xenopus*) and both flanking regions (in mouse) (amino acids in red).

The nicotinic AChR in muscle forms a heteropentamer consisting of two α-, one β-, and one δ-subunit with one γ-subunit in the fetal AChR isoform that is replaced by an ε-subunit in the adult AChR isoform (Figure 1A). All AChR subunits have a large extracellular N-terminal domain containing a signature sequence of 13 residues flanked by cysteines (C130-C144 in the human γ-subunit and C128-C142 in the human ε-subunit), four transmembrane domains (M1-M4) and a cytoplasmic loop domain of variable size and amino acid sequence between the M3 and M4 transmembrane helices (145 amino acids in the human γ-subunit and 128 amino acids in the human ε-subunit) (Figure 1B). Various functional differences between fetal and adult AChRs have been linked to structural differences between the γ- and ε-subunit (Table 1), but a generalization of the conclusions is limited by the variety of experiments performed in different species, which is important to consider especially in the context of human disease.

**TABLE 1:** Functional differences between the fetal and adult AChR.

	Fetal AChR	Adult AChR	References	Species
Subunits	α_2_βδγ	α_2_βδε		
Time of occurrence	Up to 2nd postnatal week	Thereafter	Sakmann and Brenner, 1978	Rat
	Up to 31st prenatal week, with low-level expression from sub- and perisynaptic nuclei thereafter in human	Thereafter	Hesselmans et al., 1993	Human
Location in adult muscle	Extrajunctional and junctional	Junctional	Berg et al., 1972	Rat
			Brenner et al., 1990	Rat
			Gu and Hall, 1988	Rat
Expression in extraocular muscles	En grappe endplates	En plaque endplates	Fraterman et al., 2006	Rat
	En grappe endplates	En plaque endplates	Missias et al., 1996	Mouse
	En grappe/en plaque endplates	En grappe/en plaque endplates	Kaminski et al., 1996	Rat
Conductance	40 pS	60 pS	Mishina et al., 1986	Calf
	53 pS	75 pS	Bouzat et al., 1994	Mouse
Open time	7.2 ms	2.3 ms	Mishina et al., 1986	Calf
	5.7 ms	1.6 ms	Kopta and Steinbach, 1994	Mouse
	7.9 ms	4.1 ms	Newland et al., 1995	Human
Ca^2+^ permeability	Lower	Higher	Villarroel and Sakmann, 1996	Rat
			Fucile et al., 2006	Human
			Ragozzino et al., 1998	Mouse
Resting affinity to ACh, k_*D*_	0.75 nM	22.11 nM	Nayak et al., 2014)	Mouse
Resting affinity to choline k_*D*_	1.29 μM	27.30 μM	Nayak et al., 2014	Mouse
Affinity to ^125^I-α-bungarotoxin, k_*D*_	0.04 nM	0.10 nM	Vincent et al., 1998	Human

### AChR Affinity to Acetylcholine and Channel Open Time

Each AChR has two agonist binding sites located in the extracellular domain at the interface between the principal α-subunits and the adjacent complementary γ-/ε- or δ-subunit (Figures 1A,C,D). The αγ site was shown to have a ∼35–40-fold higher affinity for acetylcholine compared with the αδ and αε sites in mice (Nayak et al., 2014), resulting in a ∼3-fold longer mean open time of fetal AChRs as compared with adult AChRs (Kopta and Steinbach, 1994). Different loops from discontinuous sections of the subunit primary sequences rearrange close to the binding sites upon agonist binding (Unwin, 2005), and a core of five aromatic amino acids from different loops were shown to be required for the stabilization of the agonist (Figures 1C,D): αY93 (loop A), αW149 (loop B), αY190 (loop C), αY198 (loop C) and γW55 (loop D) (or εW55 and δW57, respectively) (Nayak et al., 2014, 2016). At the αδ and αε sites, the free energy corresponding to the ACh affinity change in the open-channel versus resting state is mainly provided by αW149, αY190 and αY198, which together contribute −5.1 kcal/M at the αε site and −5.3 kcal/M at the αδ site (−10.4 kcal/M for αδ and αε together). At αγ, by contrast, αY93 and (especially) γW55 additionally contribute to the favorable free energy reaching a total of −7.1 kcal/M at the αγ site and of −12.4 kcal/M at the αγ site together with the αδ site (Nayak et al., 2014). However, as the core of aromatic amino acids does not differ between the three agonist binding sites, the more favorable free energy at the αγ site as compared to the αδ and αε sites must be attributed to residues in other sections of the γ-subunit, which modify the relative conformation of the aromatic core at the αγ site. Using molecular dynamic simulations and single channel electrophysiology, three residues in loop E (S111, P112, and D113) and one residue in the β5-β5′ linker (L104) near the core of the γ-subunit (C4 group) were shown to influence affinity (Figures 1C,D; Nayak et al., 2016). In constructs harboring those four residues in the δ- or ε-subunit (αδ^*C*4γ^ or αε^*C*4γ^), the exchange of the C4 group largely swapped fetal versus adult AChR affinities and open-channel lifetimes (although not accounting completely for the differences in fetal and adult AChR affinities). Further analyses revealed that the C4 group changed the aromatic core into a more compact, organized and stable conformation, with W55 now significantly contributing to AChR affinity in the αδ^*C*4γ^ and αε^*C*4γ^ constructs (Nayak et al., 2016).

The difference between fetal and adult mean open times was also reported to be determined by a 30-residue segment within the M3-M4 cytoplasmic loop at its C-terminal end as part of an amphipathic helix (Bouzat et al., 1994). In addition, two residues at the N-terminal end of the M4 domain (L458 and M460) were shown to contribute to long duration openings of fetal AChRs, and increased the mean open time of adult AChRs when introduced into the ε-subunit (Bouzat et al., 1994).

### AChR Conductance

Both AChR isoforms are selective for cations (Adams et al., 1980), the conductance of which is determined by the charge distribution along the ion permeation pathway formed by the second transmembrane (M2) domain of each of the AChR subunits (Unwin, 1995). Negatively charged residues adjacent to the extra- and intracellular portion of the pore together with the hydrophobic lining of the pore are major determinants of channel conductance (Imoto et al., 1988). In the closed AChR conformation, the hydrophobic residues of the pore prevent ion permeation by restricting water occupancy (Beckstein et al., 2001; Song and Corry, 2009). Upon AChR activation, the hydrophobic gate is relieved by widening of the pore due to a displacement of the M2 domains that allows the hydration of the pore and the conduction of ions (Miyazawa et al., 2003; Unwin and Fujiyoshi, 2012).

Adult AChRs are characterized by higher conductance (60 pS) as compared to the fetal AChR (40 pS) (Mishina et al., 1986). Using rat constructs in *Xenopus* oocytes, the exchange of the four transmembrane domains M1-M4 between γ- and ε-subunits was shown to completely reverse the difference in conductance between fetal and adult AChRs (Herlitze et al., 1996). Further analyses revealed that the major determinant of the difference in conductance was located in the M2 domains (Figure 1E), which differ at only three residues between the γ- and ε-subunit in mouse. Two of these residues (γA277 and γT278 versus εS276 and εI277) together with residues flanking the M2 domains (γK268 and γK293 versus εQ267 and εQ292) were shown to determine key differences in AChR isoform specific conductance (Herlitze et al., 1996). The significance of the M2 domain for AChR conductance was confirmed by experiments using *Xenopus* constructs, that also differed at three residues between the γ- and ε-subunit and where the exchange of two residues (γN273 and γI274 versus εS279 and εV280) contributed to a 20% decrease of the adult AChR conductance and a 22% increase of the fetal AChR conductance (Sullivan et al., 1999). As another effect of the εS279N and εV280I mutations, the mean channel open time of adult AChRs was prolonged 3.5-fold, and the γN273S mutation was associated with a 2.5-times decreased mean channel open time of the fetal AChR (with no additional effect of the γI274V mutation) (Sullivan et al., 1999). These findings were in contrast to experiments using mouse constructs that reported no contribution of the γ- and ε-subunit M2 domains to the AChR isoform specific difference in channel open times (Bouzat et al., 1994), thus revealing significant differences between amphibian and mammalian AChRs with respect to the structural determinants of fetal and adult AChR function.

### AChR Desensitization and Recovery From Desensitization

Desensitization corresponds to an accumulation of receptors in an agonist-bound but non-conducting conformational state (Sakmann et al., 1980; Colquhoun and Ogden, 1988), with a desensitization gate distinct from the activation gate (Auerbach and Akk, 1998; Wilson and Karlin, 2001) that was shown to be located at the intracellular portion of the M2 domain (Wilson and Karlin, 2001). Experiments in different models and using different applications of the patch-clamp technique revealed highly variable time constants of desensitization onset ranging from milliseconds (Paradiso and Brehm, 1998; Jahn et al., 2001; Krampfl et al., 2002) to seconds (Hopfield et al., 1988; Wagoner and Pallotta, 1988) or even minutes (Chesnut, 1983). With phosphorylation of the M3-M4 cytoplasmic AChR domains resulting in facilitation of desensitization and recovery from desensitization (Huganir et al., 1986; Hopfield et al., 1988; Hoffman et al., 1994; Paradiso and Brehm, 1998), it was possible that post-translational AChR modification together with different applications of the patch-clamp technique contributed to the varying desensitization time constants in different experimental models and between the two AChR isoforms (Auerbach and Akk, 1998; Paradiso and Brehm, 1998; Jahn et al., 2001; Krampfl et al., 2002). In a systematic analysis of desensitization and recovery from desensitization using muscle and non-muscle cell lines expressing human AChRs, recovery from desensitization was found to be faster in adult as compared to fetal human AChRs, with similar desensitization time constants between the two AChR isoforms (Cetin et al., 2019). Further analyses revealed that the M3-M4 cytoplasmic domain was a major determinant of the AChR isoform specific difference in recovery kinetics, as its exchange between the γ- and ε-subunit in chimeric constructs was sufficient to reverse recovery from desensitization between either of the AChR isoforms. Protein kinase A mediated phosphorylation of a consensus site only present in the M3-M4 cytoplasmic domain of the ε-subunit (Figure 1B) could have led to the observed difference in recovery kinetics between fetal and adult AChRs but this was not investigated further.

## Regulation of Fetal and Adult AChR Expression

### The Conversion From Fetal to Adult AChRs Is Part of the NMJ Maturation

**FIGURE 2:**
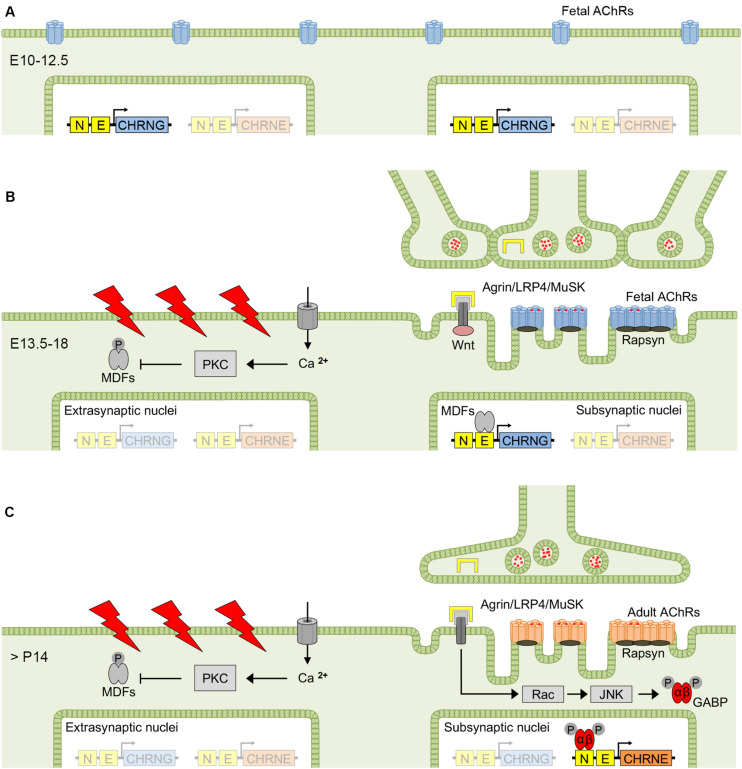
Factors determining fetal and adult AChR expression at the NMJ. Before innervation, fetal AChRs are evenly distributed at the surface of mouse myotubes **(A)**, but aggregate to clusters at the myotube center between E13.5 and E18 (i.e., prepatterning), when myotubes start to become innervated **(B)**. The exact mechanism of prepatterning is unclear but was suggested to be nerve-independent and require MuSK, LRP4 and Wnt signaling. The first 2 weeks after birth are dominated by synapse elimination resulting in singly innervated muscle fibers, and electrical activation and nerve-released agrin determine the conversion from fetal to adult AChRs, with the latter predominantly expressed in the adult muscle **(C)**. AChR, acetylcholine receptor; GABP, GA-binding protein; JNK, c-Jun NH_2_-terminal kinase; MDF, myogenic determination factor; MuSK, muscle-specific kinase; LRP4, low-density lipoprotein related protein 4; PKC, protein kinase C.

Efficient neuromuscular transmission strongly depends on the tuned coordination of structural and molecular interactions between motor neurons and muscle fibers during neuromuscular junction (NMJ) development. Rodent NMJs have been extensively studied and helped to gain insight into some of the fundamental processes involved in NMJ maturation. Soon after myoblasts fuse to form myotubes and before innervation, fetal AChRs are evenly distributed along muscle fibers (Figure 2A) and, when motor nerves reach the diaphragm in mice around embryonic day 12.5 (E12.5), the AChRs start to aggregate at the myotube center to form primitive clusters (Lin et al., 2001; Yang et al., 2001). This nerve-independent prepatterning has been suggested to be involved in axon guidance activity (Lin et al., 2008), and was shown to require the expression of postsynaptic muscle-specific kinase (MuSK) (Kim and Burden, 2008) and low-density lipoprotein related protein 4 (LRP4) (Weatherbee et al., 2006). Wnt signaling was also suggested to contribute to prepatterning in myotubes (Jing et al., 2009; Zhang et al., 2012).

Muscle innervation undergoes a substantial change during NMJ development. The period between E13.5 and E18 in mice is dominated by co-innervation of NMJs by multiple axons, most of which are eliminated in the first two postnatal weeks (Tapia et al., 2012). In a single study on human muscle, by contrast, elimination of multiple innervation occurred prenatally between the sixteenth and twenty-fifth week of gestation (Hesselmans et al., 1993). Synapse elimination in mice was associated with the disappearance of temporally correlating motor neuron activity based on the reduced gap junctional coupling of motor neurons (Personius and Balice-Gordon, 2001). As a result, asynchronous activation of the endplate region determines the elimination of the less active axonal input and, consecutively, gain of territory by the more active input (Personius et al., 2007). In rodents, the transition from multiple to single muscle fiber innervation coincides with the repression of γ-subunit mRNA and increasing ε-subunit mRNA levels transcribed from subjunctional myonuclei, whereas in human muscle, the conversion from fetal to adult AChR expression was shown to take place weeks after the transition from poly- to mononeuronal innervation (Hesselmans et al., 1993), and γ-subunit expression is maintained at low levels throughout life. Upon denervation and in human conditions that result in axonal damage (Gattenlohner et al., 2002) or lower motor neuron dysfunction such as amyotrophic lateral sclerosis (Tsujihata et al., 2001), there is an upregulation of γ-subunit expression (in slow-twitch fibers). Re-expression of the fetal AChR also occurs upon denervation in rat (Witzemann et al., 1989), mouse (Merlie et al., 1984; Goldman et al., 1988) and chicken muscle (Tsay and Schmidt, 1989), and was shown to correlate with increasing γ-subunit mRNA levels (Witzemann et al., 1989). In cases of denervation shortly after birth, the reciprocal change of γ- and ε-subunit mRNA levels is absent, and instead there is an increase of mRNA levels of all AChR subunits, with ε-subunit mRNA increasing postnatally in denervated muscle as it does in normal muscle (Witzemann et al., 1989). The generation of action potentials in the muscle fiber by activation of AChRs via nerve-released acetylcholine and the endplate potential was shown to be crucial for the regulation of AChR subunit mRNA expression (Sanes and Lichtman, 1999), with disinhibition of AChR subunit mRNA transcription in extrasynaptic nuclei upon denervation (Goldman et al., 1988) or pharmacological blockade of postsynaptic AChRs by α-bungarotoxin (Asher et al., 1991).

### Determinants of the Developmental AChR γ- to ε-Subunit Transition in Rodents

Most of the data on neuromuscular development derives from experiments with rodents, and partly chicken (Klarsfeld et al., 1989; Huang et al., 1992), and might not necessarily apply to the human situation. Therefore, this section focuses on the NMJ development in rodents, and human specific features will be outlined later in section “Developmental AChR γ- to ε-Subunit Transition in Human.”

The AChR isoform conversion is accomplished by complementary γ- and ε-subunit mRNA expression and integrates two different mechanisms that depend on motor neuron innervation. Electrical stimulation of the muscle fiber has a repressive impact on AChR gene transcription in muscle, whereas nerve-released signals increase AChR gene transcription in subsynaptic nuclei, thereby concentrating AChR surface expression to the postsynaptic membrane (Missias et al., 1996; Sanes and Lichtman, 1999; Schaeffer et al., 2001). The γ- and ε-subunit genes respond differently to these regulatory mechanisms, leading to a distinct expression pattern in subsynaptic and extrasynaptic nuclei during NMJ development (Missias et al., 1996). The γ-subunit gene is activated in all myonuclei during the initial period of myotube formation, becomes restricted to subsynaptic nuclei around the time of innervation, and disappears almost completely after birth in mice (Sakmann and Brenner, 1978). The ε-subunit, by contrast, is expressed in the adult muscle and from subsynaptic nuclei only.

Complex molecular pathways have been reported to underlie the mechanisms that control the differential γ- and ε-subunit mRNA expression, i.e., electrical muscle fiber activity and binding of motor neuron derived factors, although the exact signal cascades have not been fully unraveled yet (Tintignac et al., 2015). Depolarization of the muscle membrane by action potentials leads to an increase of cytosolic calcium resulting in activation of protein kinase C (Klarsfeld et al., 1989; Huang et al., 1992), which in turn phosphorylates and down-regulates the activity of myogenic determination factors (MDFs) (Eftimie et al., 1991; Huang et al., 1992; Figures 2B,C). MDFs comprise a number of transcriptional activators that recognize a 6 bp CANNTG motif (where N can be any nucleotide) or E-box present in the promoter region of several AChR subunit genes (Liu et al., 2000). The PKC-mediated inhibition of MDFs activity thereby results in a reduction of AChR subunit mRNA transcription in extrasynaptic nuclei. In subsynaptic nuclei, by contrast, transcription is under the control of nerve-released factors such as agrin (Jones et al., 1996). Agrin is a basal lamina proteoglycan that forms tetrameric agrin-LRP4 complexes and enhances phosphorylation and activation of MuSK (Zong et al., 2012), which in turn induces AChR subunit gene transcription via Rac-GTPase and c-Jun NH_2_-terminal kinase (JNK) (Lacazette et al., 2003; Escher et al., 2005) resulting in the phosphorylation of GA-binding protein GABPα/GABPβ dimers, both members of the Ets family of transcription factors (Schaeffer et al., 1998; Schaeffer et al., 2001). Phosphorylated GABPα/GABPβ dimers target the N-box containing the CCGGAA consensus sequence present in all AChR subunit gene promoters (Duclert et al., 1993; Koike et al., 1995; Fromm and Burden, 1998) and enhance AChR subunit gene transcription from subsynaptic nuclei (Figure 2C).

### The Time Course of AChR γ- to ε-Subunit Transition Depends on the Muscle Fiber Type

Motor units in mammalian muscle differ in their contractile properties and pattern of activity. Slow-twitch muscle fibers are innervated by motor neurons that fire in long trains of lower frequencies ranging between 10 and 20 Hz. Fast-twitch muscle fibers, by contrast, are innervated by motor neurons firing at short bursts of higher frequencies around 100 Hz (Hennig and Lomo, 1985). Immunohistochemistry of mouse muscles at different developmental stages using γ- or ε-subunit specific antibodies revealed that the subunit transition was delayed by about a week in the slow-twitch soleus muscle (with fetal AChRs still present at the NMJ up to P30) as compared to the fast-twitch extensor digitorum longus muscle (Missias et al., 1996). Their level increases in conditions associated with axonal damage and denervation in both human (Gattenlohner et al., 2002) and rodents (Kues et al., 1995; Wang et al., 2006). In human, however, the increased γ-subunit transcript levels were limited to slow-twitch fibers only (corresponding to the delayed subunit transition in slow-twitch fibers), with no up-regulation in denervated fast-twitch fibers, whereas in mouse models the upregulation of γ-subunit transcription can occur in both slow- and fast-twitch muscle fibers (Seaberg et al., 2015; Wang et al., 2016). It was thus hypothesized that γ-subunit expression is controlled by a muscle fiber type-restricted transcriptional program in human (Gattenlohner et al., 2002), the underlying mechanisms of which are yet to be revealed.

### Retention of Fetal AChR Expression Traits in Adult Extraocular Muscles

Extraocular muscles (EOMs) are characterized by specific functional and antigenic features (Kaminski et al., 1990; Yu Wai Man et al., 2005; Fraterman et al., 2006), but as mouse or rat tissue was used in most experimental studies, the data might not necessarily reflect human physiology.

As compared to limb muscles, EOMs are characterized by higher motor neuron firing rates, and consist of a mixture of both multiply innervated slow-twitch or tonic fibers and singly innervated fast-twitch fibers, with singly innervated fibers exhibiting the highest discharge frequencies (>600 Hz in monkeys; Robinson, 1970). In mice, around 80% of the EOM fibers are singly innervated and have an en plaque endplate in the muscle midbelly (Khanna et al., 2003), whereas 20% of the fibers are multiply innervated and characterized by en grappe endplates scattered along the proximal and distal end of the EOM, arising from one or more motor neurons (Porter and Baker, 1996; Khanna et al., 2003). In singly innervated fibers, firing of motor neurons generate action potentials that lead to fast-twitch contractions. Multiply innervated fibers, by contrast, have a slow-twitch or tonic mode of contraction activated focally at each synapse without the generation or propagation of action potentials (Chiarandini and Stefani, 1979). The two types of endplates differ in the transcriptional expression of AChR isoform specific subunits in rats, with ε-subunit mRNA restricted to en plaque endplates and γ-subunit mRNA to en grappe endplates (Fraterman et al., 2006). The same conclusion was made before in experiments using AChR isoform specific antibodies on mouse EOMs, with adult AChR expression restricted to en plaque endplates and fetal AChR expression to en grappe endplates (Missias et al., 1996). Yet another study on the γ- and ε-subunit immunoreactivity in rat EOMs reported contrasting results with binding of fetal and adult AChR specific antibodies to both en plaque and en grappe endplates (Kaminski et al., 1996). Several explanations were discussed to potentially underlie these discrepancies including species and age differences but also differences between global and orbital portions of EOMs (Missias et al., 1996; Fraterman et al., 2006).

Total ε-subunit mRNA levels were shown to be higher in EOMs compared to limb or intercostal muscles in human (MacLennan et al., 1997), reflecting the high density of endplates and thus high density of subsynaptic nuclei synthesizing AChR mRNAs. As AChR surface expression was not examined, however, a direct correlation of γ- or ε-subunit mRNA levels with fetal or adult AChR surface expression might be limited. The subunit conversion time course in fast-twitch singly innervated EOM fibers is similar to that of fast-twitch limb muscles with no detectable γ-subunit levels after 2 weeks postnatally in rodents (Missias et al., 1996; Fraterman et al., 2006).

### Developmental AChR γ- to ε-Subunit Transition in Human

Neuromuscular junction development has extensively been studied in rodents (Tintignac et al., 2015), revealing insight into general mechanisms of synaptic transmission. Human NMJs and AChRs share many common features with rodents, but there are also important differences that need to be acknowledged, especially in the context of human disease. The developmental AChR γ- to ε-subunit transition occurs in the second to third trimester in human (Hesselmans et al., 1993) and thus earlier than in mice, in which the transition takes place in the first 2 weeks after birth with no detectable fetal AChR expression thereafter (Sakmann and Brenner, 1978; Fischbach and Schuetze, 1980). In human, by contrast, γ-subunit mRNA continues to be expressed at low levels from sub- and perisynaptic nuclei (MacLennan et al., 1997; Croxen et al., 2001; Gattenlohner et al., 2002). This is relevant in a group of genetic disorders of the neuromuscular junction (i.e., congenital myasthenic syndrome, CMS) with underlying null mutations of the CHRNE gene coding for the ε-subunit (Croxen et al., 2001), as the persistent low-level fetal AChR expression is sufficient to enable neuromuscular transmission and patient survival. Mutant mice lacking the ε-subunit, by contrast, die within 14 weeks after birth (Missias et al., 1997).

## Significance of Fetal and Adult AChR Evolution for NMJ Function

Two whole genome duplications taking place around 500 million years ago (and before the divergence of the major vertebrate lineages) followed by gene elimination expanded a set of 10 ancestral AChR subunit genes to 16 genes in mammals including humans (Pedersen et al., 2019). As a result of the genomic duplications, a common CHRNG/CHRNE ancestor gave rise to the CHRNG and CHRNE genes in vertebrates coding for the γ- and ε-subunit, respectively. In birds, however, the CHRNE gene seems to be absent (Le Novere et al., 2002; Pedersen et al., 2019). As the CHRNB1 gene was also found to be missing in the chicken genome, it was suggested that in birds CHRNB1 and CHRNE may be located on microchromosomes that are difficult to sequence due to their GC-rich content (Pedersen et al., 2019). Alternatively, CHRNE could also have been lost in birds. This is supported by electrophysiological studies revealing that the mean AChR open time in mature chicken muscle is as long as that of fetal AChRs (Schuetze, 1980; Harvey and Van Helden, 1981) and lacks the decrease typically associated with the conversion from fetal to adult AChRs (Sakmann and Brenner, 1978). Interestingly, γ-subunit transcripts could only be detected before embryonic day 16, diminished thereafter and re-appeared only after denervation in chicken muscle tissue, suggesting the existence of another AChR subunit analogous to the ε-subunit in the adult chicken muscle (Moss et al., 1987, 1989). It was hypothesized that an AChR harboring an alternative subunit would have channel properties indistinguishable from a receptor containing the γ-subunit, or alternatively would comprise just α-, β-, and δ-subunits (Moss et al., 1987).

The γ- and ε-subunit expression in a great variety of species (Tsunoyama and Gojobori, 1998; Pedersen et al., 2019) is likely to have resulted from positive selection, as neither of the two subunits underwent gene elimination, but the physiological significance of the developmental conversion from fetal to adult AChRs remains largely unknown. Fetal AChRs are likely to be important for the prepatterning of AChR clusters in a central band during NMJ development and before innervation. In fact, using chimeric mice with a γ^ε^ -subunit (containing the C-terminal 382 amino acids of the ε-subunit) instead of the γ-subunit, prepatterning was substantially altered with formation of functional neuromuscular synapses outside the central, narrow endplate band region in the diaphragm (Koenen et al., 2005). One function of fetal AChRs could thus be to ensure a proper innervation pattern. In developing fibers, it was hypothesized that fetal AChRs may also facilitate neuromuscular transmission after innervation by allowing more charge to enter the cell during a single opening of average duration as compared to adult AChRs (Tintignac et al., 2015), which might be required for normal neuromuscular development. This is supported by experiments in rat soleus muscle, in which miniature endplate currents (mepcs) were shown to trigger spontaneous contractions of neonatal muscle fibers (expressing fetal AChRs) but not of adult muscle fibers (Jaramillo et al., 1988). The injection of both fetal- (i.e., low-amplitude and long-duration) and adult-type (i.e., high-amplitude and short-duration) mepcs into the same embryonic muscle fiber caused miniature endplate potentials of nearly the same amplitude but only fetal-type mepcs triggered action potentials. It was therefore concluded that the longer duration of fetal-type mepcs was essential for the excitation of embryonic muscle fibers. However, the spontaneous contractions elicited by fetal-type mepcs disappeared upon muscle fiber growth, which may have resulted from decreasing muscle fiber impedance rather than from the switch to the adult AChR isoform (Jaramillo et al., 1988). In another theory it was speculated that the higher sensitivity of the fetal AChR to choline could have a synergistic effect on the endplate current in developing fibers (Nayak et al., 2014), with the background level of choline shown to be especially high in fetal serum (Zeisel and Niculescu, 2006). Adult AChRs, by contrast, are characterized by faster recovery rates from desensitization (Cetin et al., 2019), and their appearance in the adult muscle could provide an adaptation to increasing motor neuron firing rates after birth, facilitating a robust and reproducible high-frequent neuromuscular transmission by preventing adult AChRs from accumulation in desensitized states. The robust high-frequent neuromuscular transmission could further be supported by the shorter mean open-channel time of adult AChRs and the faster decay of the associated endplate currents, as the depression of macroscopic currents upon repetitive stimulation was shown to depend on the particular balance between gating, entry into desensitization and the acetylcholine dissociation rate constants; this was confirmed by a study using mouse constructs, in which adult AChRs associated with faster deactivation (corresponding to a higher acetylcholine dissociation rate constant) and slower gating kinetics were less prone to desensitization (Elenes et al., 2006). And finally, the higher Ca^2+^ permeability of adult AChRs (with a fractional Ca^2+^ current of 4.2% as compared to 2.1% in the fetal isoform) could have additional physiological relevance in the regulation of neuromuscular synapse efficacy (Ragozzino et al., 1998). Transsynaptic retrograde modulation of presynaptic secretion mechanisms by the localized Ca^2+^ influx into the postsynaptic muscle cell was reported in developing *Xenopus* neuromuscular synapses (Cash et al., 1996), and autoregulatory AChR phosphorylation induced by intracellular Ca^2+^ was shown in rat myotubes (Miles et al., 1994).

## Fetal or Adult AChR Isoform Related Myasthenic Syndromes

### Myasthenia Gravis

Myasthenia gravis (MG) is an autoimmune disorder of the neuromuscular junction and mediated by autoantibodies binding to molecular targets at the postsynaptic muscle membrane. MG is clinically characterized by muscle weakness and increased fatiguability of limb, bulbar and/or extraocular muscles (Cetin and Vincent, 2018). Autoantibodies directed against the muscle AChR (AChR-Abs) can be detected in about 80% of patients with generalized MG and 50% of patients with MG restricted to the EOMs (ocular MG) (Lindstrom et al., 1976; Vincent and Newsom-Davis, 1985). There is no clear evidence for a correlation of AChR-Ab titers with disease severity between individuals (Vincent and Newsom Davis, 1980; Roses et al., 1981), although ocular MG patients tend to have lower AChR-Ab concentrations (Vincent and Newsom Davis, 1980). Within an individual undergoing treatment, however, there is usually a good correlation between the fall in antibody levels and clinical improvement; both reversing if the treatment (e.g., plasma exchange) is stopped (Newsom-Davis et al., 1979).

The AChR-Abs appear to be polyclonal and heterogenous even in individual patients and were shown to target the extracellular domains of all five subunits including the γ- or ε-subunit (Vincent and Newsom-Davis, 1979; Vincent et al., 1987; Tzartos et al., 1998; Kostelidou et al., 2007; Zisimopoulou et al., 2008), with a relatively high but variable proportion of the antibodies binding to the main immunogenic region on the two α-subunits (Tzartos et al., 1982; Whiting et al., 1986). Antibodies with preferential binding to the γ- or the ε-subunit can more often be found in ocular MG patients (Vincent and Newsom-Davis, 1982; MacLennan et al., 1997; Shi et al., 2012). Using cell-based assays expressing fetal or adult AChRs, the proportion of MG patients with γ- or ε-subunit specific AChR-Abs was higher in ocular MG (12 and 14%, respectively) as compared to generalized MG (2 and 7%, respectively) (Shi et al., 2012). Using radioimmunoprecipitation assays, AChR-Abs from ocular MG patients were shown to react better with adult AChRs (Vincent and Newsom-Davis, 1982; MacLennan et al., 1997), in contrast to the earlier suggestion that the preferential involvement of the EOMs in ocular MG patients was a result of fetal AChR expression by these muscles (Horton et al., 1993; Oda, 1993). The higher susceptibility of EOMs in MG was instead ascribed to a lower safety factor of singly innervated fast-twitch fibers resulting from less prominent postsynaptic folds with fewer AChRs on the postsynaptic membrane (Kaminski et al., 1990; Yu Wai Man et al., 2005). Moreover, women with very high levels of antibodies that inhibit the ion channel activity of fetal AChRs, but not adult AChRs (see below), do not have predominant ocular symptoms.

Higher motor neuron firing frequencies could further contribute to a lower safety factor by increasing the release of acetylcholine from the presynaptic terminal sufficiently to overcome the catalytic activity of the acetylcholine esterase, thus leading to prolongation of the lifetime of acetylcholine in the synaptic cleft and accumulation of AChRs in desensitized states (Magleby and Pallotta, 1981; Ruzzier and Scuka, 1986; Giniatullin et al., 1993). Additionally, prolonged high frequency vesicle release can lead to depletion of presynaptic vesicles with consequent reduction of the quantal content (Kaminski et al., 1990). In multiply innervated tonic fibers, on the other hand, contraction depends directly on the depolarization of the postsynaptic membrane induced by the endplate current rather than a sodium channel-dependent action potential (Kaminski et al., 1990; Yu Wai Man et al., 2005). EOMs also differ immunologically from limb muscles in that complement regulatory genes are expressed in lower levels that may contribute to the susceptibility of EOMs to MG (Porter et al., 2001; Kaminski et al., 2004), with preferential involvement of EOMs at lower AChR-Ab titers (Vincent and Newsom Davis, 1980).

### Neonatal Myasthenic Syndromes Due to Placental Transfer of Maternal AChR-Abs

Neonates born to mothers with MG can sometimes develop transient neonatal MG (TNMG) due to placental transfer of AChR-IgG-Abs (Papazian, 1992; Vernet-Der Garabedian et al., 1994). In cases with restriction of fetal movement *in utero* during the second trimester, the clinical picture is characterized by multiple joint contractures or skeletal deformities such as scoliosis and lung hypoplasia. This condition is termed arthrogryposis multiplex congenita (AMC) and often associated with the requirement of the neonates for assisted ventilation or with prenatal or neonatal death (Vincent et al., 1995). AMC is sometimes classified as the most severe form of the fetal acetylcholine receptor inactivation syndrome (FARIS), which sometimes follows TNMG with the development of a persistent myopathy predominantly affecting facial and bulbar muscles, hearing loss, pyloric stenosis and central nervous system involvement in early childhood (Oskoui et al., 2008; Hacohen et al., 2015; Allen et al., 2016). Mothers of myasthenic neonates can have clinically manifest MG, and the treatment of those mothers after diagnosis can lead to the subsequent birth of healthy babies. In babies with AMC, however, some mothers are asymptomatic with unrecognized disease (Barnes et al., 1995; Vincent et al., 1995; Riemersma et al., 1996), suggesting that the AChR-Abs are different from those usually found in MG. In fact, AChR-Abs in AMC (and FARIS) preferably bind to fetal AChRs (Vincent et al., 1995; Hacohen et al., 2015), whereas AChR-Abs crossing the placenta in TNMG commonly bind to both fetal and adult AChRs via the shared α-subunits (Vernet-Der Garabedian et al., 1994). Fetal AChR-Abs were also more common in women who developed MG after pregnancy as compared with women who presented before pregnancy, and were therefore hypothesized to be induced by the fetus (Matthews et al., 2002). These antibodies were shown to inhibit fetal but not adult AChR function (Vincent et al., 1995), with 80% current reduction within 2 min application of serum from mothers of neonates born with AMC (Cetin et al., 2020). When injected into pregnant mice, a proportion of the mouse embryos showed fixed joint deformities, were paralyzed or stillborn (Jacobson et al., 1999), confirming the pathogenic role of the fetal AChR-Abs.

### CHRNG Associated Congenital Myasthenic Syndrome

Mutations of the AChR γ-subunit gene CHRNG have been found to underlie Escobar type multiple pterygium syndrome (Hoffmann et al., 2006; Morgan et al., 2006), an autosomal recessive form of AMC characterized by webbing (pterygia), congenital fixed joint deformities and scoliosis (Escobar et al., 1978; Rajab et al., 2005). Respiratory distress, intrauterine death and stillbirths are common. The developmental abnormalities result from the truncation or low expression of the γ-subunit, with loss of fetal AChR function at sensitive times of neuromuscular development (Murray and Drachman, 1969; Hammond and Donnenfeld, 1995). The AChR subunits have to be assembled in the endoplasmic reticulum before the receptor can be inserted into the plasma membrane, starting with the formation of an αβγ trimer and sequential addition of a δ- and a second α-subunit (Green and Claudio, 1993; Wanamaker et al., 2003). If the γ-subunit is missing, the receptor cannot reach the cell surface resulting in AChR deficiency (Hoffmann et al., 2006). Patients usually show little or no progression of their condition and no myasthenic symptoms after birth, as neuromuscular transmission continues to be mediated by the adult AChR, although they may display residual developmental abnormalities such as webbing of the neck and between the fingers. The survival of some cases in which the γ-subunit is missing due to truncating mutations within the N-terminal region would indicate at least some early low-level expression of the ε-subunit (Hesselmans et al., 1993).

### CHRNE Associated Congenital Myasthenic Syndrome

Mutations of the AChR ε-subunit gene CHRNE are the most frequent and depending on the ethnic population may account for over 30% of cases with congenital myasthenic syndromes (Engel et al., 2003, 2015; Finlayson et al., 2013). Mutations are found along the entire gene including the promoter region (Nichols et al., 1999; Engel et al., 2015) and result in protein truncation, missense mutation of essential residues or in reduced levels of ε-subunit mRNA expression. The defects are heterogeneous and include AChR deficiency, slow-channel, fast-channel and low-conductance syndromes (Ohno et al., 1997; Webster et al., 2012; Rodriguez Cruz et al., 2018). The high prevalence of CHRNE mutations has been attributed to the phenotypic rescue by the persistent low-level expression of the γ-subunit that is sufficient to enable patients with ε-subunit null alleles to survive (Croxen et al., 2001; Cossins et al., 2004). The phenotypic spectrum of CHRNE associated primary AChR deficiency is variable and ranges from mild to severe, but most patients are characterized by marked ophthalmoparesis (Engel et al., 1996; Burke et al., 2004; Rodriguez Cruz et al., 2015), which could be related to the exceptionally high motor neuron firing frequencies of extraocular muscles (Yu Wai Man et al., 2005), requiring fast recovery rates from AChR desensitization for efficient neuromuscular transmission. Neuromuscular transmission in patients with CHRNE null mutations, however, has to depend on the fetal AChR with slow recovery rates from desensitization. Fetal AChRs could accrue into desensitized states and might be unable to promote efficient neuromuscular transmission at high motor neuron firing frequencies (Cetin et al., 2019).

## Concluding Remarks

The transition from fetal to adult AChR expression during NMJ development represents a characteristic feature in mammals with complex regulatory mechanisms mediated by the fine-tuned electrical and molecular communication between nerve and muscle. Intensive research of the past decades produced insight into functional differences between both AChR isoforms and has helped to understand better their physiological significance and their role in different myasthenic syndromes. However, some key aspects of fetal and adult AChR function remain to be answered. Especially the elucidation of the structural determinants of desensitization and recovery from desensitization in fetal and adult AChRs and their evaluation in animal models could further help to unravel the significance of desensitization in NMJ physiology and disease.

## Author Contributions

HC drafted the manuscript and prepared the figures. DB, AV, and RW edited and reviewed the manuscript. All authors contributed to the article and approved the submitted version.

## Conflict of Interest

The authors declare that the research was conducted in the absence of any commercial or financial relationships that could be construed as a potential conflict of interest.
